# Mechanisms and Future Research Perspectives on Mitochondrial Diseases Associated with Isoleucyl-tRNA Synthetase Gene Mutations

**DOI:** 10.3390/genes15070894

**Published:** 2024-07-08

**Authors:** Masaki Watanabe, Nobuya Sasaki

**Affiliations:** Laboratory of Laboratory Animal Science and Medicine, Kitasato University, 35-1, Higashi-23, Towada 034-8628, Aomori, Japan

**Keywords:** aminoacyl-tRNA, IARS1, IARS2, mitochondrial disease

## Abstract

Aminoacyl-tRNA synthetases are essential enzymes for the accurate translation of genetic information. IARS1 and IARS2 are isoleucyl-tRNA synthetases functioning in the cytoplasm and mitochondria, respectively, with genetic mutations in these enzymes causing diverse clinical phenotypes in specific organs and tissues. Mutations in *IARS1* and *IARS2* have recently been linked to mitochondrial diseases. This review aims to explore the relationship between IARS1 and IARS2 and these diseases, providing a comprehensive overview of their association with mitochondrial diseases. Mutations in *IARS1* cause weak calf syndrome in cattle and mitochondrial diseases in humans, leading to growth retardation and liver dysfunction. Mutations in *IARS2* are associated with Leigh syndrome, craniosynostosis and abnormal genitalia syndrome. Future research is expected to involve genetic analysis of a larger number of patients, identifying new mutations in *IARS1* and *IARS2*, and elucidating their impact on mitochondrial function. Additionally, genetically modified mice and the corresponding phenotypic analysis will serve as powerful tools for understanding the functions of these gene products and unraveling disease mechanisms. This will likely promote the development of new therapies and preventive measures.

## 1. Introduction

Aminoacyl-tRNA synthetases (aaRSs) are essential enzymes that link nucleic acids and proteins, ensuring the accurate translation of genetic information [1,2]. By catalyzing the esterification reaction that attaches transfer RNA (tRNA) to its corresponding amino acid, aaRSs ensure that the correct amino acid corresponds to the anticodon of the tRNA [3,4]. Additionally, mitochondria possess their own genetic information and maintain a unique protein synthesis system [5]. This is based on the endosymbiotic theory, which suggests that mitochondria were once free-living bacteria that were incorporated into primitive eukaryotic cells and formed a symbiotic relationship [6]. As a result, mitochondria have their own DNA and maintain an independent protein synthesis mechanism, including their own ribosomes and tRNA [7]. Due to this background, there is a necessity for mitochondrial aaRSs [8]. Mitochondria use their own tRNA and ribosomes to synthesize proteins encoded by their own DNA. Therefore, mitochondria require specialized aaRSs adapted to their system [9]. There are structural differences between cytoplasmic and mitochondrial tRNA, necessitating specialized aaRSs for each [10]. These differences ensure the precise attachment of amino acids. Additionally, cytoplasmic aaRSs and mitochondrial aaRSs each fulfill different roles in different locations, maintaining efficient overall cellular function [11]. In prokaryotes, such as Escherichia coli, there is usually one type of aaRS. In contrast, eukaryotes have two types of aaRSs, a cytoplasmic type and a mitochondrial type, which function in the cytoplasm and mitochondria, respectively. This differentiation and maintenance are important evolutionary adaptations that support the complex regulation and efficient function of protein synthesis in eukaryotic cells [12,13]. For most other amino acids as well, there are both cytoplasmic and mitochondrial types of aaRSs [14]. This necessity arises from the requirement to synthesize each amino acid within the mitochondria, and each aaRS must correctly bind to its specific tRNA and attach the corresponding amino acid accurately, ensuring precise protein synthesis [15].

**Figure 1:**
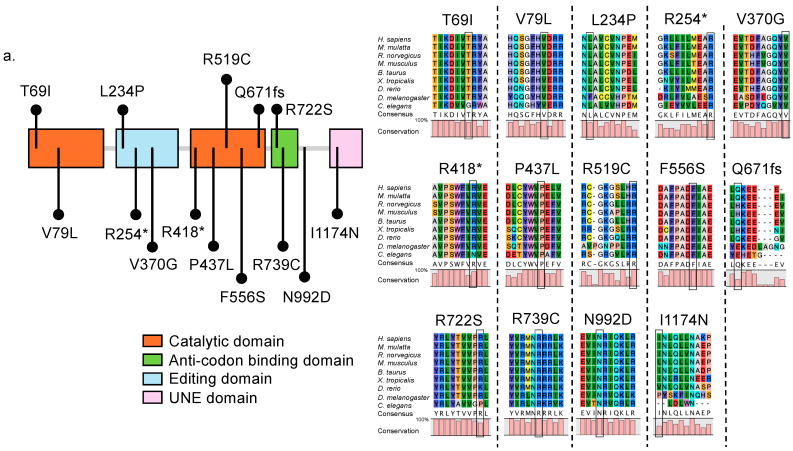

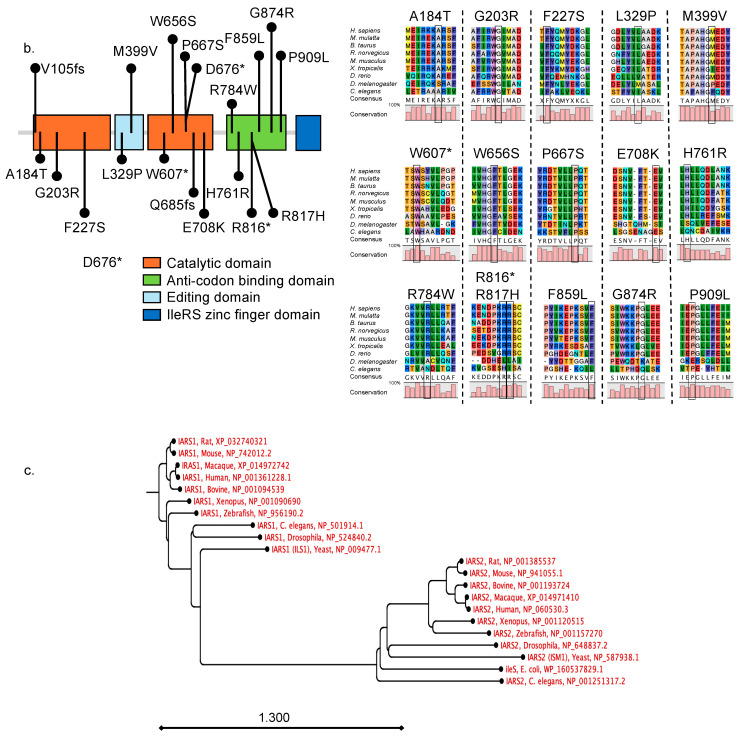
IARS1 and IARS2 scheme, sequence alignment and homology model. (**a**) Schematic view of IARS1 protein and sequence alignment (*Homo sapiens* (*H. sapiens*), NP_060530.3; *Macaca mulatta* (*M. mulatta*), XP_014972742; *Bos taurus* (*B. taurus*), NP_001094539; *Mus musculus* (*M. musculus*), NP_198653.2; *Rattus norvegicus* (*R. novegicus*), XP_032740321; *Xenopus tropicalis* (*X. tropicalis*), NM_001127043.1; *Danio rerio* (*D. rerio*), XM_021467083.1; *Drosophila melanogaster* (*D. melanogaster*), AAO41285; *Caenorhabditis elegans* (*C. elegans*), NP_50191.4). (**b**) Schematic view of IARS2 protein and sequence alignment. The bar graph below the amino acid alignment indicates the degree of conservation between sequences as a percentage. Asterisks indicate termination codons. (**c**) Phylogenetic tree of IARS orthologous proteins. We collected IARS protein sequences from different species using NCBI, along with their accession numbers. Using the neighbor-joining method, we constructed a phylogenetic tree to understand their evolutionary history. The tree is drawn to scale, with branch lengths representing the number of amino acid differences per site. These differences were calculated using the p-distance method. The numbers next to the branches indicate how often the species grouped together in 1000 tests. The length of the scale bar represents the average number of substitutions per site. All evolutionary analyses were performed using CLC Sequence Viewer 8.

IARS1, an isoleucyl-tRNA synthetase (IleRS), contains crucial peptide sequences involved in amino acid activation, such as the catalytic domain, important sequences for recognizing the appropriate tRNA molecules, such as the anticodon-binding domain (Figure 1a), and several highly conserved domains: (a) the IleRS core domain, which functions as the catalytic core for the aminoacylation reaction, binding isoleucine to tRNA, and is highly conserved across different species with identical functions confirmed in yeast, zebrafish, and humans; (b) the editing domain, which is related to nucleotide transfer and exhibits a high degree of conservation across different species, indicating its evolutionarily important role; and (c) the anticodon-binding domain, which recognizes the anticodon of the tRNA and ensures that the correct amino acid is attached, and is also highly conserved and essential for accurate protein synthesis [16]. IARS2, another IleRS, similarly contains conserved, functionally important domains, including (a) the tRNA synthetase domain, which features the HIGH motif and the KMSKS motif; (b) the anticodon-binding domain, which ensures the correct amino acid is attached by recognizing the anticodon of tRNA; and (c) the FPG IleRS zinc finger domain, which contributes to the structural stability of the protein (Figure 1b) [17]. Alignment analysis indicates that both the cytoplasmic IleRS IARS1 and the mitochondrial IleRS IARS2 are derived from a common ancestral gene, with IARS2 being closer to the ancestor of prokaryotes (Figure 1c).

In recent years, mutations in both alleles of *IARS1* and *IARS2* have been linked to mitochondrial diseases. Therefore, this review aims to summarize the findings on the association between *IARS1* and *IARS2* mutations and these diseases.

## 2. Overview of Basic Biological Functions of IARS

**Figure 2:**
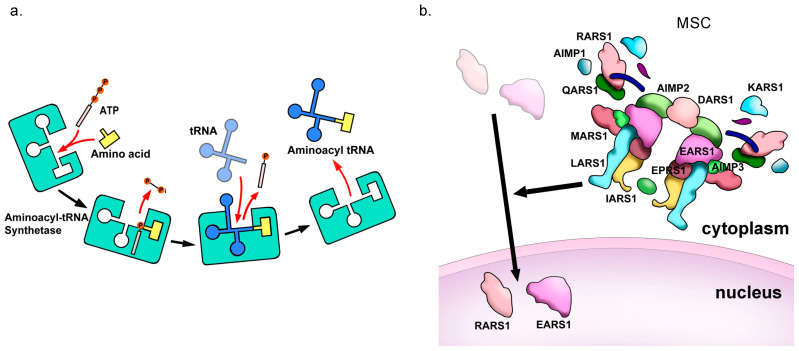
(**a**) Overview of the esterification reaction, catalyzed by aaRS proteins. (**b**) Roles of aminoacyl-tRNA synthetase-interacting multi-functional protein.

aaRSs are essential enzymes expressed in all cells, responsible for charging tRNAs with their corresponding amino acids, which is a crucial step in translating genetic information into proteins [11]. aaRSs charge tRNAs through two steps: (i) using ATP to activate the amino acid, forming an aminoacyl-adenylate intermediate; and (ii) transferring the aminoacyl group to the tRNA, releasing the charged tRNA (Figure 2a) [18]. Additionally, nine aaRSs [arginyl (RARS1), aspartyl (DARS1), glutaminyl (QARS1), glutamyl-prolyl (EPRS1), isoleucyl (IARS1), leucyl (LARS1), lysyl (KARS1), and methionyl-tRNA synthetase 1 (MARS1)], and three auxiliary proteins [ARS-interacting multifunctional proteins: AIMP1 (p43), AIMP2 (p38), and AIMP3 (p18)] form a multi-enzyme complex that regulates functions beyond translation by controlling the intracellular localization of aaRSs (Figure 2b) [19]. IARS2, on the other hand, is the mitochondrial IleRS encoded by nuclear DNA. Protein translation within the mitochondria requires an apparatus different from that of cytoplasmic translation in eukaryotic cells, and IARS2 catalyzes the synthesis of isoleucyl-tRNA necessary for synthesizing proteins encoded by mitochondrial DNA (mtDNA) [20]. In addition to its conventional tRNA aminoacylation activity, the IARS1 enzyme possesses various non-canonical functions. Bowen et al. showed that increased expression of *IARS1* promotes the phenotypic transformation of vascular smooth muscle cells to a synthetic type via activation of the p38 MAPK signaling pathway. Additionally, increased expression of *IARS1* induces apoptosis of vascular smooth muscle cells through the downregulation of the PI3K signaling pathway, resulting in cellular dysfunction and contributing to the development of abdominal aortic aneurysms [21]. Bing-Xi Yan et al. showed that, in a mouse model of imiquimod-induced psoriasis-like dermatitis, mupirocin suppresses the expression of *IARS*, thereby inhibiting epidermal proliferation, the expression of inflammatory cytokines and chemokines, and the infiltration of immune cells. This effect is mediated by inhibition of the IL-17 signaling pathway [22]. Chung et al. showed that IARS1 interacts with other proteins via its C-terminal UBX domain, protecting these proteins from degradation through ubiquitination and thus stabilizing them. Furthermore, IARS1 interacts with BRCA1 in the nucleus, maintaining the stability of BRCA1 and participating in DNA repair, particularly homologous recombination repair [23]. Although *BRCA1* mutations are associated with multiple types of cancer [24], there are no reports on the involvement of *IARS1* mutations in carcinogenesis. However, IARS1 plays an important role in DNA repair and the maintenance of chromosomal stability through BRCA1, suggesting that its deficiency or dysfunction may contribute to carcinogenesis. Furthermore, because *IARS1*-deficient cells become more sensitive to PARP inhibitors, *IARS1* is being considered a potential target for cancer therapy [23].

## 3. Mitochondrial Disease

Mitochondrial diseases are genetic disorders caused by mitochondrial dysfunction [25,26,27]. It is estimated that 1 in 5000 newborns [28,29] and 1 in 8000 adults [30] have mitochondrial genetic disorders. Mitochondria are organelles within cells that play a crucial role in energy production. As a result, mitochondrial diseases exhibit a wide range of symptoms and unique challenges in diagnosis and treatment. First, the symptoms of mitochondrial diseases are highly diverse because mitochondria are present in all cells of the body, affecting multiple organs and tissues such as muscles, the nervous system, the heart, the kidneys, and the endocrine system [25,26,27]. Second, the diagnosis of mitochondrial diseases is difficult because of their clinical and genetic diversity. Over 300 genes have been identified with causative mutations, and this number continues to increase [31]. Mitochondrial diseases are caused by mutations in either mtDNA or nuclear DNA. In children, 15% to 30% of cases result from mtDNA mutations, whereas in adults, more than 50% are caused by mtDNA mutations, often inherited from the mother [32]. The age of onset varies from the neonatal period to adulthood, and the severity of symptoms in individual patients also varies widely, from mild to severe. Owing to the diversity of symptoms, diagnosis can be challenging, and genetic testing or muscle biopsy may be required [33,34]. Currently, there is no cure for mitochondrial diseases, and there are no Food and Drug Administration (FDA)-approved treatments. This lack of treatment options results from the different genes and phenotypes associated with these diseases. A combination of vitamins, cofactors, nutrients, and antioxidants, known as the “mitochondrial cocktail”, has been suggested to alleviate symptoms, limit disease progression, and overcome mitochondrial toxicity [29,35]. Mutations in *IARS1* are particularly associated with mitochondrial hepatopathy, characterized by liver dysfunction accompanied by mitochondrial impairment [16]. Mitochondrial hepatopathy primarily manifests itself in the neonatal or infantile period because of genetic abnormalities in the mitochondrial respiratory chain. Symptoms are non-specific and include neonatal hyperammonemia, neonatal liver failure, and infantile liver failure [36,37]. It is estimated that mitochondrial hepatopathy occurs in approximately 20% of patients with mitochondrial disease, accounting for approximately 5–10% of neonatal hyperammonemia cases, 10–20% of neonatal liver failure cases, and 10–20% of infantile liver failure cases [38,39]. Various genes are responsible for this condition, and mutations in genes involved in the replication and maintenance of mtDNA, such as *DGUOK*, *MPV17*, *SUCLG1*, *POLG1*, *QIL1*, and *TWNK*, lead to defective mtDNA synthesis or abnormal reductions in mtDNA. Consequently, dysfunction of the respiratory chain complex and increased oxidative stress lead to mitochondrial hepatopathy [40,41,42,43,44,45]. Similar to *IARS2*, mutations in other mitochondrial aaRS genes, such as *LARS*, *FARS2*, and *WARS2*, also cause mitochondrial hepatopathy [46,47,48]. However, there are no reports on mitochondrial hepatopathy in *IARS2* deficiency. For diagnosis, measuring serum biomarkers and assessing respiratory chain enzyme activity through liver biopsy are useful, but genetic testing is necessary for a definitive diagnosis [49].

In recent years, advancements in omics technologies have significantly propelled mitochondrial medicine forward. First, in the field of genomics, the introduction of next-generation sequencers improved the diagnostic rate of mitochondrial diseases by more than 50%, leading to the discovery of numerous novel causative genes [50,51]. Second, in the field of transcriptomics, microarray-based gene expression profiling has contributed to a better understanding of the pathology of mitochondrial diseases. For example, profiling of a mouse model with mutations in the Twinkle helicase and patient-derived transmitochondrial cybrids bearing the common m.3243A>G mtDNA mutation, associated with MELAS syndrome, identified new biomarkers such as fibroblast growth factor 21 (FGF21) and growth and differentiation factor 15 (GDF15) [52,53]. In the field of proteomics, comprehensive analyses of mitochondrial proteins have revealed the structure and function of respiratory chain complexes and identified new assembly factors [54,55]. Furthermore, in the field of metabolomics, several metabolites with altered levels in mitochondrial diseases have been identified, suggesting new pathological mechanisms such as one-carbon metabolism, which includes folate and methionine metabolism [56,57]. These omics analyses have revealed that mitochondria function dynamically as a central hub of metabolism and signaling. New therapeutic approaches are also being evaluated, such as redox-regulating drugs, nucleic acid supplementation therapy, and mitochondrial genome editing [58,59,60,61].

## 4. Involvement of *IARS1* in Weak Calf Syndrome

The phenotypes resulting from *IARS1* mutations were first observed in cattle. Calves with *IARS* abnormalities exhibit weak calf syndrome, characterized by low birth weight, anemia, irregular body temperature, susceptibility to infections, thymic atrophy, and fatty liver from an early age. Physical characteristics include slender limbs, thin thighs, loose joints, skull deformities, and a thin throat. These calves, from a few days to approximately three months old, are weak, have poor appetite despite standing and nursing, are small and thin with a narrow body width, and have loose skin. Additionally, they are prone to infections, diarrhea, pneumonia, and otitis media [62,63]. Hirano et al. conducted genetic analysis in a specific bull lineage with a higher than usual incidence of weak calf syndrome and neonatal mortality. Homozygosity mapping and exome analysis identified a single SNP (c.235G>C, p.Val79Leu) in the IARS1 gene located on the bovine chromosome 8 as the causative mutation. The detected mutation (c.235G>C, p.Val79Leu) reduces the enzymatic activity to less than 40% [63]. On the other hand, there have been no reports of mitochondrial dysfunction caused by *IARS1* mutation in weak calf syndrome. Therefore, further research is necessary to understand the impact on mitochondrial function. The authors created genetically modified mice with the bovine variant of the mutation to successfully replicate the pathophysiology of IARS disorders. In *Iars1* V79L mutant mice, a significant increase in serum ornithine carbamoyltransferase levels was observed, suggesting the presence of mitochondrial hepatopathy. Proteomic analysis revealed that the mitochondrial function-related protein NME4 was significantly downregulated in IARS1 V79L mutant mice [64]. The reduction in NME4 disrupts the balance of mitochondrial fission and fusion, which is crucial for maintaining mitochondrial structure and function. This imbalance leads to decreased ATP production, compromising cellular energy metabolism. Additionally, the reduction in NME4 increases the production of reactive oxygen species (ROS), which can damage the mitochondrial membrane and DNA [65,66]. These factors collectively impair mitochondrial function, leading to broader cellular dysfunction and potentially contributing to various mitochondrial diseases. Additionally, a decrease in the expression of proteins involved in liver lipid metabolism, such as AK6, JAK3, LILRB3, and UBASH3B, was also observed. This study suggests that the loss of IARS function leads to mitochondrial dysfunction and may directly contribute to fatty liver pathogenesis [64].

## 5. Involvement of *IARS1* in Mitochondrial Diseases

Following the discovery of IARS1 abnormalities owing to *IARS1* mutations in cattle, Kopajtich et al. identified *IARS1* mutations in a human family with mitochondrial disease [16]. Abnormalities in the human *IARS1* gene cause symptoms such as intrauterine growth retardation, liver dysfunction (fatty liver) from infancy, growth disorders including short stature, hearing loss, and diabetes. The breakdown of symptoms includes postnatal growth retardation (nine out of nine patients), intellectual disability (eight out of nine patients), zinc deficiency (five out of nine patients), hypotonia (seven out of nine patients), hepatic steatosis (four out of nine), liver fibrosis (four out of nine patients), abnormal liver function (seven out of nine) and diabetes/hearing loss (one out of nine patients) [16,67,68,69,70]. A decrease in mitochondrial respiratory chain enzyme activity is observed in fibroblasts, muscles, and liver, indicating that these symptoms result from decreased activity of these enzymes. Specifically, in Japanese patients, fibroblasts showed reduced activity in respiratory chain enzyme complexes I and IV (37% and 41% of citrate synthase ratio, respectively). In German patients, muscles exhibit reduced activity in respiratory chain enzyme complexes I, IV, and pyruvate dehydrogenase (77%, 52%, and 64% of normal control, respectively). In Austrian patients, a decrease in respiratory chain enzyme complex I activity was observed (46% of normal control) [16]. Mitochondrial function has not been investigated in patients from Israel, Poland, and China. The functional impairment of the identified *IARS1* mutations was confirmed through complementation assays in yeast and functional analyses in zebrafish. Kopajtich et al. analyzed six mutations identified in patients (p.Arg418*, p.Ile1174Asn, p.Arg254*, p.Pro437Leu, p.Val370Gly, and p.Asn992Asp). Strains expressing the two nonsense mutations (p.Arg418*, p.Arg254*) and the p.Asn992Asp mutation failed to grow similarly to those with the empty vector. Strains expressing the four missense mutations (p.Val79Leu, p.Ile1174Asn, p.Pro437Leu, p.Val370Gly) showed reduced growth compared to the wild-type. In zebrafish, *IARS1* knockdown resulted in overall developmental delay, brain malformation, and significant shortening of the posterior body axis [16]. Additionally, Orenstein et al. transformed an *IARS1*-deficient yeast strain with wild-type *IARS1*, mutant *IARS*, or the pYY1 plasmid without an insert. Cells expressing the p.Phe556Ser mutant showed weak growth after four days, with a marked reduction in colony number and size compared to the wild-type. Cells expressing the p.Arg739Cys mutant did not grow at all after either two or four days. These results indicate that the p.Phe556Ser mutant is functionally impaired in vivo, whereas the p.Arg739Cys mutant is functionally a null allele [69]. Jiang et al. discovered new *IARS1* gene mutations in a 17-month-old female patient from China. Genetic analysis revealed the presence of two novel *IARS1* mutations in the patient. One mutation, c.120-1G>A, was inherited from the father and is a splice-site mutation. The other, c.2164C>A (p.Arg722Ser), was inherited from the mother and is a missense mutation. According to SpliceAI predictions, the c.120-1G>A mutation leads to the loss of the splice acceptor site, causing exon skipping and resulting in premature transcription termination. The p.Arg722Ser mutation is located in the anticodon-binding domain and has been shown to result in loss of function in yeast complementation assays [70] (Table 1).

## 6. Involvement of *IARS2* in Mitochondrial Diseases

Mitochondrial aaRSs play a crucial role in attaching amino acids to tRNAs required for protein synthesis within the mitochondria. When these enzymes become dysfunctional, the corresponding amino acids cannot accurately bind to tRNAs, hindering protein synthesis. Mitochondria synthesize essential proteins, such as those in the oxidative phosphorylation (OXPHOS) complexes, based on their own DNA [72,73]. If the synthesis of these proteins is disrupted, it severely impacts energy production. Mutations in *IARS2* are associated with mitochondrial diseases characterized primarily by Leigh syndrome and craniosynostosis and abnormal genitalia syndrome (CAGSSS). Leigh syndrome is a mitochondrial disease in which organs and tissues that require a high amount of ATP, such as the brain, heart, skeletal muscles, and liver, are primarily affected. Symptoms include elevated lactate levels in the blood and/or cerebrospinal fluid, developmental delay, respiratory disorders, epileptic seizures, feeding difficulties, and muscle weakness [74,75]. Neuroimaging with CT or MRI typically reveals lesions in the basal ganglia, thalamus, brainstem, cerebellum, and posterior columns of the spinal cord. These lesions appear as low density on CT scans, high signal intensity on T2-weighted MRI, and low signal intensity on T1-weighted MRI, which results from disrupted electron transport chain complexes caused by mutations in mtDNA or nuclear DNA [76,77]. CAGSSS is characterized by cataracts, growth hormone deficiency, sensory neuropathy, sensorineural hearing loss, and skeletal dysplasia [78]. Mutations in the *IARS2* gene were first reported to be associated with CAGSSS in a large French-Canadian family. Three probands from this family were homozygous for a pathogenic missense mutation (c.2726C>T, p.Pro909Leu) in exon 21 of the 23-exon *IARS2* gene. This proline substitution affects the predicted anticodon-binding domain [78]. Furthermore, a Danish proband also exhibited CAGSSS symptoms and had a homozygous pathogenic mutation (c.2620C>A, p.Gly874Arg) affecting the same exon 21 [79]. Another proband with compound heterozygous mutations (c.1821G>A, p.Trp607*; c.2122G>A, p.Glu708Lys) was diagnosed with Leigh syndrome and died at 18 months of age [78]. In two Chinese probands, compound heterozygous *IARS2* mutations associated with sporadic pediatric cataracts (c.607G>C, p.Gly203Arg and c.2575T>C, p.Phe859Leu; c.2446C>T, p.Arg816* and c.2575T>C, p.Phe859Leu) were found, representing the mildest clinical presentation among IARS2-related disorders [80]. In 2018, two Japanese siblings were reported with novel compound missense mutations (c.680T>C, p.Phe227Ser and c.2450G>A, p.Arg817His) showing mild symptoms of CAGSSS and West syndrome combined with Leigh syndrome [81]. Additionally, a new homozygous missense mutation (c.2282A>G, p.His761Arg) was identified in an Iranian family, presenting clinical features limited to cataracts and skeletal dysplasia [17]. In 2024, a 14-month-old Chinese girl presented with West syndrome and Leigh syndrome. Whole-exome sequencing identified genetic mutations, revealing c.2450G>A (Arg817His) and a copy number variation [g. (220267549_220284289) del] [82].

In addition to these neurological and developmental manifestations, *IARS2* mutations have significant cardiac and hematologic implications. Cardiomyopathy (both dilated and hypertrophic), conduction abnormalities, and ventricular dysfunction are typical cardiovascular manifestations. Upadia et al. reported a female with *IARS2* compound heterozygous variants, p.Val499fs and p.Arg784Trp who presented Wolff–Parkinson–White pattern, characterized by an abnormal extra electrical pathway in the heart, leading to episodes of rapid heart rate [83].

Furthermore, IARS2-related disorders can present with sideroblastic anemia, a condition where the bone marrow produces ringed sideroblasts rather than healthy red blood cells, leading to ineffective erythropoiesis. Sideroblastic anemia was reported in seven current cases, including one where the patient exhibited resistance to standard treatments and required frequent blood transfusions. The anemia was resistant to high-dose intravenous γ globulin and cyclosporine therapy, necessitating monthly blood transfusions to maintain hemoglobin levels [83,84,85]. These findings highlight the broad and complex phenotypic spectrum of IARS2-related diseases, encompassing not only neurological and developmental issues but also significant cardiac and hematologic complications (Table 2). The pathogenic mechanism of mitochondrial diseases caused by *IARS2* mutations is primarily related to impaired protein synthesis within the mitochondria, but the details remain largely unknown.

## 7. Discussion and Future Perspectives

Although IARS1 is not directly localized in the mitochondria, it enhances protein synthesis efficiency in the cytoplasm and regulates DNA repair [23]. IARS1 is also involved in the downregulation of the mitochondrial function-related protein NME4 [64]. The reduction in IARS1 expression induces the production of ROS, leading to mitochondrial oxidative stress and damage. For instance, excessive generation of ROS can damage the mitochondrial membrane and DNA, resulting in decreased mitochondrial function [86]. Cytoplasmic aaRSs play a crucial role in overall cellular energy metabolism. Dysfunction of aaRSs affects ATP production and metabolic pathways, which, in turn, impacts mitochondrial energy metabolism [87]. Since mitochondria are the primary energy-producing organelles in the cell, this has serious consequences for overall cellular function. Due to these diverse functions, IARS1 is believed to indirectly contribute to mitochondrial function and play a crucial role in maintaining overall cellular function. On the other hand, IARS2 is localized in mitochondria and directly facilitates protein synthesis within the organelle. When mutated, it leads to mitochondrial dysfunction due to aberrant translation of the OXPHOS respiratory chain and subsequent production of ROS [79]. However, in diseases associated with mitochondrial aaRSs, immortalized fibroblast cell lines from patients do not show a correlation between the severity of cases and the deficiency of OXPHOS complexes, complicating recapitulation studies and leaving details unclear [88]. Diseases caused by mutations in *IARS1* and *IARS2* exhibit mitochondrial dysfunction that affects various organs, showing some similarities. However, *IARS1* mutation is primarily characterized by mitochondrial hepatopathy, whereas *IARS2* mutation is associated with CAGSSS, indicating different prevalent sites and suggesting significant differences in the disease mechanisms between IARS1-related and IARS2-related disorders.

Future research is expected to involve extensive genetic analysis using a larger patient population. This will help identify new mutations in *IARS1* and *IARS2* and clarify the impact of these mutations on mitochondrial function. Detailed mechanistic studies on how *IARS1* and *IARS2* mutations lead to mitochondrial dysfunction are particularly needed, including evaluating their effects on mitochondrial protein translation, assembly, and function. Developing therapies for *IARS*-related mitochondrial diseases is also a critical challenge, encompassing gene therapy, enzyme replacement therapy, and drugs that support mitochondrial function. Furthermore, early diagnosis and the establishment of effective management strategies are expected to improve patients’ quality of life. Discovering new biomarkers and developing clinical guidelines to delay symptom progression will contribute to this goal. Additionally, research on IARS-related mitochondrial diseases is expected to accelerate through international cooperation and data sharing, advancing knowledge and the development of treatments for rare diseases. These future perspectives aim to deepen the understanding of mitochondrial diseases associated with *IARS1* and *IARS2* mutations and contribute to the development of future therapies. Moreover, phenotypic analysis of genetically modified mice and omics analysis of affected organs are powerful tools for understanding the function of gene products and elucidating disease mechanisms, promoting the development of new therapies and preventive measures. Genetically modified mice also accelerate the development of new treatments. Researchers can conduct preclinical trials using these models to assess the efficacy of new drugs or therapeutic approaches targeting mitochondrial dysfunction. This is especially crucial given the limited treatment options currently available for mitochondrial diseases. These models contribute to elucidating disease mechanisms at cellular and molecular levels. They facilitate the identification of cellular signals and metabolic pathways involved in mitochondrial diseases, potentially leading to the discovery of novel therapeutic targets. Comparing IARS mutant mice with normal mice allows for detailed studies on how mitochondrial diseases affect biological functions, thereby enhancing our understanding of gene functions and disease mechanisms. Overall, the use of genetically modified mice with IARS mutations is extremely valuable in mitochondrial disease research. These models deepen our understanding of the diseases and expedite the development of new treatments. As research in this field progresses, it is expected to enhance our comprehension of IARS-related mitochondrial diseases and lead to the establishment of effective therapeutic strategies.

## Figures and Tables

**Table 1:** Genetic and clinical findings in patients with IARS1 mutations.

Proband	Sex	IARS1 Mutations	Clinical Features						Ref.
		cDNA; Protein	Growth Retardation	Intellectual Disability	Infantile Hepatopathy	Zinc Deficiency	Diabetes Melitus	Recurrent Infection	
German	M	c.[1252C>T], [3521T>A]; p.[Arg418*], [lle1174Asn]	+	+	+	+	−	+	[16]
Japanese	F	c.[760C>T], [1310C>T]; p.[Arg254*], [Pro437Leu]	+	+	+	+	+	−	[16]
Austrian	M	c.[1109T>G], [2974A>G]; p.[Val370Gly], [Asn992Asp]	+	+	+	+	−	+	[16]
Israeli Arab	M	c.[2215C>T], [1667T>C]; p.[Arg739Cys], [Phe556Ser]	+	+	+	−	−	−	[69]
Polish	M	c.[2011delC], [206C>T]; p.[Gln671fs], [Thr69lle]	+	+	+	+	−	+	[67]
Chinese	F	c.[1305G>C], [3377dup]; p.[Trp435Cys], [Asn1126fs]	+	ND	+	−	−	−	[71]
Chinese	F	c.[701T>C], c.[1555C>T]; p.[Leu234Pro], p.[Arg519Cys]	+	+	+	−	−	+	[68]
Chinese	F	c.[2164C>A], c.[120-1G>A]; p.[Arg722Ser]	+	+	+	+	−	+	[70]
Bovine	M/F	c.[235G > C], c.[235G > C]; p.[Val79Leu], p.[Val79Leu]	+	ND	+	−	−	+	[63]

Asterisks indicate termination codons.

**Table 2:** Genetic and clinical findings in patients with IARS2 mutations.

Proband	Sex	IARS2 Mutations	Clinical Features				Ref.
		cDNA; Protein	CAGSSS	Leigh Syndrome	Cataract	Sideroblastic Anemia	
French-Canadian	M/F	c.[2726C > T], [2726C > T]; p.[Pro909Leu], [Pro909Leu]	+	−	+	−	[78]
Scandinavian	M	c.[1821G>A], [2122G>A]; p.[Trp607*], [Glu708Lys]	−	+	−	−	[78]
Danish	F	c.[2620C > A], [2620C > A]; p.[Gly874Arg], [Gly874Arg]	+	−	−	−	[79]
Japanese	F	c.[680T>C], [2450G>A]; p.[Phe227Ser], [Arg817His]	+	+	+	−	[81]
Iran	M	c.[2752C>T], [2752C>T]; p.[Pro909Ser], [Pro909Ser]	+	−	+	−	[17]
Iran	F	c.[2282A>G], [2282A>G]; p.[His761Arg], [His761Arg]	+	−	+	−	[17]
Chinese	M	c.[607G>C], [2575T>C]; p.[Gly203Arg], [p.Phe859Leu]	−	−	+	−	[80]
Chinese	M	c.[2446C>T], [2575T>C]; p.[Arg816*], [Phe859Leu]	−	−	+	−	[80]
Korean	M	c.[314_318del], [2450G>A]; p.[Val105fs], [Arg817His]	−	+	−	−	[86]
Korean	F	c.[1195A>G], [2052del]; p.[Met399Val], [Gln685fs]	−	+	−	−	[86]
Korean	F	c.[550G>A], [1967T>C]; p.[Ala184Thr], [Phe656Ser]	−	+	−	−	[86]
Korean	M	c.[971_972del], [2450G>A]; p.[Ser324*], [Arg817His]	−	+	−	−	[86]
French	M	c.[891G>A], [2450G>A]; p.[Trp297*], [Arg817His]	+	−	+	+	[84]
French	M/F	c.[2025dup], [986T>C]; p.[Asp676*], [Leu329Pro]	+	−	+	−	[84]
Sri Lankan	M	c.[199C>T], [199C>T]; p.[Pro67Ser], [Pro67Ser]	−	−	+	+	[84]
African-American	F	c.[1493dup], [2350C>T]; p.[Val499fs], [Arg784Trp]	+	−	−	−	[83]
African-American	F	c.[2450G>A], [2511del]; p.[Arg817His], [Leu838fs]	−	−	+	+	[85]
Chinese	F	c.[2450G>A], g. (220267549_220284289) del; p.[Arg817His]	−	+	−	+	[82]

Asterisks indicate termination codons.

## Data Availability

No new data were created or analyzed in this study. Data sharing is not applicable to this article.
